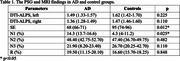# Polysomnographic findings and DTI‐ALPS in Alzheimer's disease

**DOI:** 10.1002/alz70856_101623

**Published:** 2025-12-24

**Authors:** Lusine A. Brsikyan, Ekaterina V. Vasileva, Kseniya V. Nevzorova, Anastasiia N. Sergeeva, Marina V. Krotenkova, Amayak G. Broutian, Ekaterina Yu. Fedotova, Sergey N. Illarioshkin

**Affiliations:** ^1^ Research Center of Neurology, Moscow, Russian Federation; ^2^ Research Center of Neurology, Moscow, Other, Russian Federation

## Abstract

**Background:**

Sleep disorders are common comorbid pathology in patients with Alzheimer's disease (AD). It is generally accepted that sleep disorders may be a risk factor for AD development due to increased Aβ deposition in the brain and may contribute to more rapid progression of cognitive deficit. Increased deposition of Aβ occurs mainly as a result of disruption of the glymphatic system, which functions most effectively during slow‐wave sleep. The diffusion tensor images analysis along perivascular spaces (DTI‐ALPS) method is used for indirect assessment of the glymphatic system. In our study we compare polysomnographic (PSG) findings, DTI‐ALPS indices in AD and controls, and identify the relationship between these indicators.

**Method:**

The study included 2 groups: 7 patients with the amnestic variant of AD (6 females, mean age 69 years) and 7 cognitively intact volunteers (5 females, mean age 61 years). All participants underwent brain MRI with evaluation of DTI‐ALPS indices, PSG with determination of sleep efficiency (SE) and sleep staging (N1, N2, N3, R).

**Result:**

A statistically significant decreased SE and the increased duration of stage N1 (%) in AD were revealed. A tendency to a decrease in the duration of stage N3 (%), DTI‐ALPS indices in AD is also noted, however statistically insignificant (Table 1). A correlation analysis of MRI and PSG findings revealed a significant inverse relationship between the right DTI‐ALPS index and the duration of stage N1 (%) (*p* = 0.016*), between the left DTI‐ALPS index and the duration of stage N2 (%) (*p* = 0.027*). Additionally, we found a tendency to a moderate direct relationship between the duration of stage N3 (%) and the right DTI‐ALPS index (*p* = 0.089) as well as the left DTI‐ALPS index (*p* = 0.081).

**Conclusion:**

In AD patients the PSG findings of disturbed sleep were detected. The revealed correlations suggested possible relationship between altered sleep architecture and insufficient glymphatic system functioning in neurodegeneration that requires further study.